# Caveolin-1 in sarcomas: friend or foe?

**DOI:** 10.18632/oncotarget.255

**Published:** 2011-04-02

**Authors:** Miguel Sáinz-Jaspeado, Juan Martin-Liberal, Laura Lagares-Tena, Silvia Mateo-Lozano, Xavier Garcia del Muro, Oscar M Tirado

**Affiliations:** ^1^ Sarcoma Research Group, IDIBELL (Bellvitge Biomedical Research Institute), L'Hospitalet de Llobregat, Barcelona, Spain; ^2^ Nanomedicine Research Program, Molecular Biology and Biochemistry Research Center, CIBBIM-Nanomedicine, Vall d'Hebron Hospital Research Institute, Barcelona, Spain

**Keywords:** Caveolin-1, Sarcomas, Oncogene, Tumor suppressor

## Abstract

Sarcomas represent a heterogeneous group of tumors with a complex and difficult reproducible classification. Their pathogenesis is poorly understood and there are few effective treatment options for advanced disease. Caveolin-1 is a multifunctional scaffolding protein with multiple binding partners that regulates multiple cancer-associated processes including cellular transformation, tumor growth, cell death and survival, multidrug resistance, angiogenesis, cell migration and metastasis. However, ambiguous roles have been ascribed to caveolin-1 in signal transduction and cancer, including sarcomas. In particular, evidence indicating that caveolin-1 function is cell context dependent has been repeatedly reported. Caveolin-1 appears to act as a tumor suppressor protein at early stages of cancer progression. In contrast, a growing body of evidence indicates that caveolin-1 is up-regulated in several multidrug-resistant and metastatic cancer cell lines and human tumor specimens. This review is focused on the role of caveolin-1 in several soft tissue and bone sarcomas and discusses the use of this protein as a potential diagnostic and prognostic marker and as a therapeutic target.

## INTRODUCTION

Sarcomas constitute a heterogeneous category of neoplasms composed mostly of uncommon tumors of different histology, biology, and outcome. According to its molecular features, soft-tissue and bone sarcomas can be classified in two big groups: associated to specific genetic alterations or without specific molecular patterns [1]. One third of sarcomas have well defined genetic alterations. The identification of these genetic patterns has supposed a revolution since some of them have diagnosis, prognosis and therapeutic implications [2]. They are further divided in two particular categories: sarcomas with specific chromosomal translocations and sarcomas with specific gene mutations [3].

About 15-20% of sarcomas are associated with specific chromosomal translocations involving, most commonly, a member of the TET family (EWS, FUS or TAF15) and a transcription factor. These translocations are thought to happen early in carcinogenesis, promoting some of the processes that finally lead to the appearance of cancer cells [4]. Single gene mutations in certain group of sarcomas encode proteins leading to tumor formation. Usually, the product derived from the mutated gene is a transmembrane tyrosine kinase receptor constitutively activated in a ligand-independent manner. This activation triggers intracellular pathways that finally lead to carcinogenesis [5].

About two thirds of sarcomas lack specific genetic alterations. Adult sarcomas frequently belong to this group and complex karyotypes are often found instead of chromosomal translocations. In this category of sarcomas, p53 inactivation seems to be an early and common event in carcinogenesis. Anomalies in the retinoblastoma pathway are also frequently found but no specific patterns of abnormalities can be described in this subgroup of tumors [6]. Thus, tumors with the same diagnosis present different molecular patterns and different chromosomal aberrations depending on each case.

## CAVEOLIN-1

**FIGURE 1:**
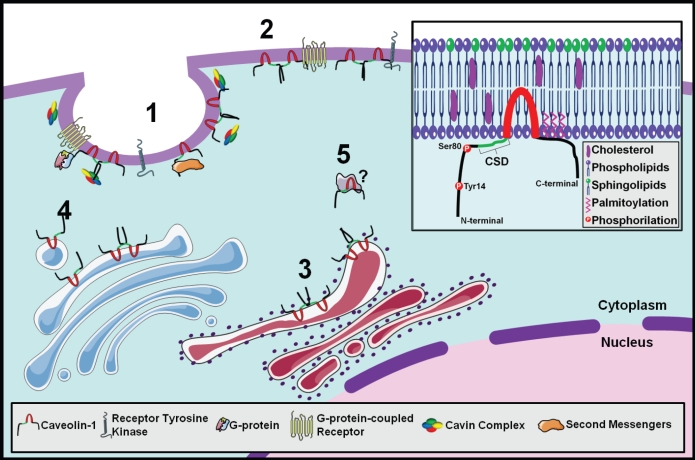
CAV1 localization options inside the cell **1** In caveolae complexed with cavins interacting with tyrosine-kinase or G-protein coupled receptors. **2** Out of caveolae interacting with the same receptors. **3** At the endoplasmic reticulum synthesis of CAV1. **4** From the plasma membrane to the golgi apparatus caveolae-dependent endocytic functions. **5** In the cytoplasm interacting with still unknown proteins. **Inset** Structure and membrane topology of CAV1 showing phosphorylation sites, the caveolin scaffolding domain (CSD), and the transmembrane domain.

Caveolin-1 (CAV1) belongs to a family of proteins named caveolins. There are three members: CAV1, 2 and 3, which can form homo-and hetero-oligomeric complexes mediated by domains in the N- and C-terminal domains [7]. CAV1 and 2 are ubiquitously expressed in a variety of cell types such as endothelial cells, pneumocytes, adipocytes and fibroblasts [8]. Expression of CAV2 nearly always mirrors that of CAV1. This may be, in part, due to requirement of CAV1 to transport CAV2 to the plasma membrane where it can be incorporated into caveolae [9]. In contrast, expression of CAV3 is restricted to striated muscle cells and is a component of the sarcoplasmic reticulum of skeletal, cardiac and smooth muscle [10, 11]. CAV1 is the major structural protein in caveolae; small invaginations within the plasma membrane. Caveolae are involved in signal transduction, wherein CAV1 acts as a scaffold to organize multiple molecular complexes regulating a variety of cellular events, for a complete review see [12]. However, CAV1 might be present on flat plasma membrane or/and on different organelles (Figure 1). Whether its role is different at such diverse locations remains to be elucidated. Proteins that associate with CAV1 contain the canonical caveolin-1 binding domain, фxфxxxф or фxxxxфxxф (where ф= Trp, Phe or Tyr). Interaction with a large majority of these proteins occurs through the caveolin scaffolding domain (CSD) (Figure 1). It is the ability to interact with numerous proteins that makes CAV1 a keystone in signaling by organizing signaling complexes at the inner plasma membrane [12].

CAV1 has been shown to possess an ambiguous role in cancer and to act both as a tumor suppressor or promoter. Both activities had been described for CAV1 in breast, oral, colon, lung, uterus and thyroid cancer. This apparent contradiction may be explained by different interacting partners during cancer progression [13], and it is proposed that *in vivo*, CAV1 plays a tissue and stage specific modulatory role in cancer [14].

## CAV1 IN SARCOMAS

**FIGURE 2:**
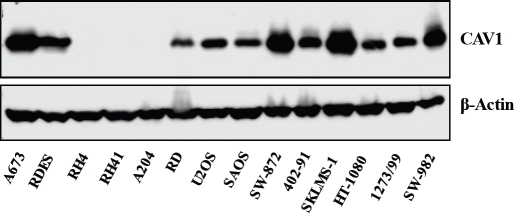
Western Blot analysis showing CAV1 expression in different sarcoma cell lines: Ewing Sarcoma (A673, RDES); Alveolar Rhabdomyosarcoma (Rh4, RH41); Embryonal Rhabdomyosarcoma (A204, RD); Osteosarcoma (U2OS, SAOS); Liposarcoma (SW-872); Myxoid Liposarcoma (402-91); Leiomyosarcoma (SKLMS-1); Fibrosarcoma (HT-1080); Synovial Sarcoma (1273/99, SW-982)

Because CAV1 is most abundantly expressed in terminally differentiated mesenchymal cells such as smooth muscle cells, adipocytes and endothelial cells, Wiechen et al. investigated whether it was a tumor suppressor in sarcomas [15]. By immunohistochemistry they found that CAV1 expression was high in fibroblasts, smooth muscle cells, adipocytes and endothelial cells with a fine granular membranous and a diffuse cytoplasmic staining pattern. Moreover, levels of CAV1, comparable to normal mesenchymal cells, were retained in all benign mesenchymal tumors, including 5 of 5 fibromatoses, 7 of 7 leiomyomas, 4 of 4 lipomas, and 6 of 6 hemangiomas. CAV1 expression was found to be absent or strongly reduced in 3 of 3 fibrosarcomas, 17 of 20 leiomyosarcomas, 5 of 8 angiosarcomas, 15 of 18 malignant fibrous histiocytomas, and 8 of 8 synovial sarcomas. Therefore it was concluded that CAV1 is a candidate tumor suppressor gene in sarcomas [15]. Accordingly, the analysis of CAV1 in Gastrointestinal stromal tumors (GISTs) suggested that this protein may also act as a tumor suppressor [16]. The authors showed that in a sample of 108 GISTs patients 86.1% (93 patients) did not express CAV1. However, there was no correlation between the caveolin-1 expression status and any of the clinicopathologic variables, including mitosis and tumor grade. The expression of caveolin-1 was not correlated with other immunohistochemical marker proteins including, c-kit, CD34 and SMA. On the univariate analysis, CAV1 expression was not a significant predictor of the disease-free survival for GIST patients [16]. Other studies focusing in the expression of CAV1 in specific sarcomas have shown that this protein is expressed in the cytoplasm of cells from a variety of mesenchymal benign tumors, including the adipocyte of all types of lipoma and well-differentiated liposarcoma, the myocyte of angiomyolipoma, leiomyoma, and well-differentiated leiomyosarcoma [17]. The immunostaining properties were uniform among the cells of each of these lesions, and gender and age did not influence the results. However, all of the malignant mesenchymal tumors which are poorly differentiated and dedifferentiated, including leiomyosarcoma and liposarcoma, showed weak immunoreactivity or failed to stain with CAV1 [17], suggesting that loss of CAV1 might be a necessary step towards a differentiation block typical of malignancy and visible in most soft tissue sarcomas. Interestingly, we have observed high expression of CAV1 in some cell lines representative of these sarcomas, such as leiomyosarcoma and synovial sarcoma (Figure 2). Therefore, whether the expression of CAV1 in these cells correlates with the degree of differentiation and consequently it relates to any function, requires further investigation. Nevertheless, there are some functional studies about CAV1 in specific sarcomas such Ewing's sarcoma family of tumors (ESFT), Osteosarcoma and Rhabdomyosarcoma trying to figure out the clinical importance of this protein that we will review thereafter:

### Ewing's sarcoma family of tumors

ESFT includes aggressive bone-associated malignancies that affect the pediatric population. Nearly all ESFT patients already have micrometastases at diagnosis, resulting in a >95% relapse rate when treated locally and a 40% relapse rate after systemic chemotherapy. Most ESFT harbor a reciprocal translocation, t(11;22)(q24;q12), which links a strong transcriptional activation domain from EWS to the ETS DNA-binding domain of the transcription factor FLI-1 [18]. The *EWS/FLI-1* fusion is required for Ewing's sarcoma oncogenesis, as inhibition of its function results in the loss of transformation of ESFT cells [19-23]. CAV1 was identified as a metastasis-associated gene that is a transcriptional target of EWS/FLI-1 as well as an important determinant of ESFT malignant phenotype and tumorigenicity [24]. Using antisense and short hairpin RNA-mediated gene expression knockdown, array analyses, chromatin immunoprecipitation methods, and reexpression studies, the authors showed that CAV1 is a new direct target of EWS/FLI-1 that is overexpressed in ESFT cell lines and tumor specimens and is necessary for ESFT tumorigenesis. CAV1 knockdown led to up-regulation of Snail and the concomitant loss of E-cadherin expression. Consistently, loss of CAV1 expression inhibited the anchorage-independent growth of EWS cells and markedly reduced the growth of Ewing's sarcoma cell-derived tumors in nude mice xenografts, indicating that CAV1 promotes the malignant phenotype in Ewing's sarcoma carcinogenesis. Reexpression of CAV1 or E-cadherin in CAV1 knockdown Ewing's sarcoma cells rescued the oncogenic phenotype of the original Ewing's sarcoma cells, showing that the CAV1/Snail/E-cadherin pathway plays a central role in the expression of the oncogenic transformation functions of EWS/FLI-1 [24]. Later on, CAV1 with other 3 proteins was considered a differential diagnostic immunomarker for Ewing's sarcoma/PNET in a sample of 415 genetically confirmed cases [25].

Another study from the same authors showed that CAV1 expression determines the sensitivity of ESFT cells to clinically relevant chemotherapeutic agents [26]. Analyses of endogenous CAV1 levels in several ESFT cells and ectopic CAV1 expression into ESFT cells expressing low endogenous CAV1 showed that the higher the CAV1 levels, the greater their resistance to drug treatment. Moreover, results from antisense- and shRNA-mediated gene expression knockdown and protein re-expression experiments demonstrated that CAV1 increases the resistance of ESFT cells to doxorubicin- and cisplatin-induced apoptosis by a mechanism involving the activating phosphorylation of PKCalpha. CAV1 knockdown in ESFT cells led to decreased phospho-PKCalpha levels and a concomitant sensitization to apoptosis, which were reversed by CAV1 re-expression. These results were recapitulated by PKCalpha knockdown and re-expression in ESFT cells in which CAV1 was previously knocked down, thus demonstrating that phospho-PKCalpha acts downstream of CAV1 to determine the sensitivity of ESFT cells to chemotherapeutic drugs. These data, along with the finding that CAV1 and phospho-PKCalpha are co-expressed in approximately 45% of ESFT specimens tested [26], implied that targeting CAV1 and/or PKCalpha may allow the development of new molecular therapeutic strategies to improve the treatment outcome for patients with ESFT.

Our group has demonstrated that CAV1 controls migration and invasion in ESFT cells in culture by mechanisms involving the production and activation of metalloproteinases as well as lung colonization in nude mice by regulating SPARC expression levels [27], adding relevance to the key roles that CAV1 plays in ESFT biology. Moreover, by ectopic expression of a Myc-tagged CAV1 protein in ESFT cells, as well as the supplementation of culture media with purified CAV1 protein followed by its intracellular localization using immunofluorescence, we showed that ESFT cells secrete CAV1. Likewise, we showed that ESFT cells are able to take up the secreted protein, and that extracellular CAV1 enhances EWS cell proliferation [28]. Whether this secreted CAV1 has roles other than proliferation remains to be elucidated.

### Osteosarcoma

Osteosarcoma (OS) is the most common primary tumor of bone, occurring predominantly in the second decade of life. High-dose cytotoxic chemotherapy and surgical resection have improved prognosis, with long-term survival for patients with localized (non-metastatic) disease approaching 70%. At presentation approximately 20% of patients have metastases and almost all patients with recurrent OS have metastatic disease, and cure rates for patients with metastatic or recurrent disease remain poor [29]. CAV1 has been shown to act as an oncosuppressor in human osteosarcoma; its down-regulation is part of osteoblast transformation and osteosarcoma progression [30]. In the study the authors did a survey of 6-year follow-up that indicated a better overall survival for osteosarcoma expressing a level of CAV1 similar to osteoblasts. Moreover, the majority of primary osteosarcoma showed significantly lower levels of CAV1 than normal osteoblasts suggesting its role as an oncosuppressor. Mechanistically, the authors showed that Met-induced osteoblast transformation was associated with CAV1 down-regulation. *In vitro*, osteosarcoma cell lines forced to overexpress CAV1 showed reduced malignancy with inhibited anchorage-independent growth, migration and invasion. *In vivo*, CAV1 overexpression abrogated the metastatic ability of osteosarcoma cells. They also showed that c-Src and c-Met tyrosine kinases, which are activated in osteosarcoma, co-localized with CAV1 and were inhibited upon CAV1 overexpression [31]. In contrast, in a recent immunohistochemical study of 61 xenotransplanted osteosarcoma tumors it was shown that CAV1 showed immunoreactivity in the majority of the tumors with no significant variation among the subtypes or subsequent passages; even in the majority of the metastatic cases. Nevertheless, the authors could not conclude that CAV1 is a marker either for good or for bad prognosis [31], suggesting that the role of CAV1 in OS requires further investigation.

### Rhabdomyosarcoma

Rhabdomyosarcoma, a neoplasm composed of skeletal myoblast-like cells, represents the most common soft tissue sarcoma in children. It can be divided into two major histological subtypes: so-called embryonal and alveolar rhabdomyosarcoma [32]. The embryonal subtype is the most common and predominates at favorable anatomic sites such as the orbit, other head and neck sites, and the genitourinary tract. The alveolar subtype occurs in both children and adults, and it is more common at extremity sites and carries an overall inferior prognosis [32]. Rhabdomyosarcoma is defined histologically as a small round blue cell tumor which expresses markers of myogenic differentiation, such as MyoD, myogenin, desmin, and actin. These myogenic markers discriminate it from other soft tissue or bone sarcomas, but late markers of myogenic differentiation are absent, and rhabdomyosarcoma cells do not form myotubes or functional muscle units [33]. Alveolar rhabdomyosarcoma is associated in the vast majority of cases with a specific balanced translocation involving chromosomes 2 and 13 [t(2;13)] or, less commonly, 1 and 13 [t(1;13)] [34, 35], each of which encodes a novel fusion protein, PAX3/FOXO1 and PAX7/FOXO1, respectively. In skeletal muscle, CAV1 and Caveolin-3 (CAV3), a member of the caveolin family expressed specifically in muscle tissue, are both expressed. In particular, CAV1 expression is restricted to satellite cells, which represent a pool of quiescent reserve elements; whereas CAV3 is expressed in myoblasts undergoing differentiation and in mature fibers [36, 11], suggesting that a timely coordinated expression of CAV1 and CAV3 contributes to skeletal muscle homeostasis. In rhabdomyosarcomas, CAV3 is considered a sensitive and specific marker of both subtypes [37]. In contrast to other sarcomas, CAV1 has been suggested to be a marker of poor differentiation for rhabdomyosarcomas [38] associating its expression to a better prognostic entity. CAV1 was predominantly expressed in the embryonal subtype and its expression was associated to an immature cell phenotype. On the other hand, most alveolar rhabdomyosarcoma tumors exhibiting advanced degree of maturation had very low levels of CAV1 suggesting that CAV1 might be a tumor suppressor in alveolar rhabdomyosarcomas. However, the number of samples used in this study was very small and further analysis using a bigger sample would be highly desirable.

## PERSPECTIVE

In the recent years it has been relatively well agreed that depending on tumor type, CAV1 can have either tumor suppressor or oncogenic effects on a cell. Many proteins contain putative CAV1 binding domains. The effect of CAV1 on tumor phenotype seems to be very heterogeneous and strongly dependent on the molecular partners interacting with this protein [13]. Several important proteins involved in cell transformation and growth have been shown to interact with CAV1 including the molecules that stimulate tumor cell invasion and cytoskeletal rearrangement such as growth factor receptors, protein kinases, heterotrimeric G-proteins and Rho GTPases [39-42]. In general, CAV1 is supposed to concentrate signaling molecules within specialized membrane domains, named caveolae [43, 44]. In several cell lines, despite the high expression levels of CAV1, caveolae are not morphologically distinguishable suggesting a non-caveolar role for CAV1. For example, CAV1 can accumulate at focal adhesions and as reported by several groups, this translocation requires CAV1 phosphorylation at Tyr14. Thus, this might represent a novel example of a caveolae-independent role of CAV1 as a molecular organizer. Also, there is no evidence supporting the existence of caveolae-like invaginations in neurons however, physical organization between CAV1 and several receptors may occur in the context of membrane microdomains [45]. Furthermore, it is now clear that cavins (a new family of proteins that form the cavin complex) (Figure 1) are indispensable for caveolae formation and function [46]. Therefore, the possible loss of cavins in tumor cells opens a new exciting window to explore caveolar and non-caveolar roles of CAV1.

In epithelial tumors the role of CAV1 in sarcomas is very contradictory; this is aggravated by the fact that functional studies are very scarce. However, as demonstrated in some bone and soft tissue sarcomas, CAV1 may have a key role in their malignant progression. From our point of view it will be of key importance to correlate caveolar and non-caveolar functions with tumor suppressor or oncogenic activities.

Depending on the role assigned to CAV1 in every sarcoma type it will be possible to use different targeting options. In those sarcomas where CAV1 was demonstrated to act as a tumor suppressor, targeted ectopic re-expression or introduction of a CSD would be a feasible option. In fact, re-expression of CAV1 has successfully reduced the tumor growth of breast cancer cells [47] and the invasive capability of pancreatic [48] and breast [49] cancer cells. Delivery of CSD peptides fused to the C-terminus of the Drosophila antennapedia (AP) homeodomain internalization sequence to subcutaneously implanted tumors inhibited tumor progression by reducing vascular permeability and mediating an indirect anti-angiogenic effect [50]. On the other hand, direct targeting of CAV1 using antisense and siRNA, or indirectly by chemical inhibition, or lowering cholesterol (disrupts caveolae) may result of great help in the cases where CAV1 acts as an oncogene. Similar to prostate cancer [51] and melanomas [52], our group successfully showed that targeting CAV1 with shRNAs reduced Ewing's sarcoma progression [27]. In multiple myeloma, proteasome inhibitors blocked VEGF-triggered CAV1 phosphorylation and expression resulting in reduced migration and survival [53]. The use of statins to inhibit cholesterol synthesis in glioma cells suppressed CAV1 expression and consequently reduced migration and survival [54]. Whether these drugs may be of use for the treatment of sarcoma patients remains unknown. However, the development of new reagents such as siRNAs, peptide and small-molecule inhibitors will define new avenues for therapies, not only for some sarcomas but for tumors that present CAV1-triggered progression. In addition, as more protein and signaling molecules are found to interact with and regulate CAV1 expression the design of novel therapies for sarcomas will evolve. These therapies may include humanized antibodies, small-molecule inhibitors and targeted siRNAs altogether with improved gene therapy delivery systems.
